# Effects of Movement for High Time-Bandwidths in Batched Pulse Compression Range-Doppler Radar

**DOI:** 10.3390/s21072492

**Published:** 2021-04-03

**Authors:** Dominik Bok, Daniel O’Hagan, Peter Knott

**Affiliations:** 1 Fraunhofer FHR, Fraunhofer Institute for High Frequency Physics and Radar Techniques FHR, Fraunhoferstr. 20, 53343 Wachtberg, Germany; daniel.ohagan@fhr.fraunhofer.de (D.O.); peter.knott@fhr.fraunhofer.de (P.K.); 2 Institute of High Frequency Technology, RWTH Aachen University, Melatener Str. 25, 52074 Aachen, Germany

**Keywords:** range walk, Doppler processing, Doppler spread, range compression, integration gain

## Abstract

Radar detection and track building performance is an essential part of a radar system. A high realized coherent integration gain often contributes to an improved performance. This is essential to the successful detection and tracking of weak moving targets. However, the actual movement within the coherent processing interval can introduce range walk effects. The processing will then result in range and Doppler frequency resolutions that become finer than a single moving point scatterer’s spread over range and—often not considered—over Doppler frequency. In particular for a wide instantaneous bandwidth, the impact on the achievable integration gain can become severe already for a constant effective velocity. Therefore, high desired integration gains as required in passive radar are not easily achieved against relatively fast moving targets. The main intent of this article is to present the movement effects on a classical range-Doppler analysis to give an insight on the achievable performance and to quantify otherwise appearing degradations. Interestingly, a classical analysis of experimental datasets evaluated from a DVB-T based passive radar measurement campaign even resolved the fluctuation of a target response within the instantaneously processed bandwidth. The findings strengthen the need for advanced processing methods that can at least partly address individual implications of fast moving targets in real-time applications properly.

## 1. Introduction

The capability of a radar to track a target depends strongly on its ability to detect moving targets with good signal level margins. More energy retrieved from an object usually comes along with a better detection performance. The range-Doppler analysis enables hereby the simultaneous determination of range and target velocity that is effectively measurable from the radar node. This information is then used to localise the target and to estimate further movements to establish a track. Despite most objects to be detected are actually moving, most radar processing schemes assume inherently a constant effective velocity and that the target movement can be fully addressed by a single Doppler frequency displacement. As long as this is a valid model, this principle will enhance the radar detection and tracking performance against moving objects. This assumption is obviously limited by range migration and a closer analysis turns out that in fact the time-bandwidth product is crucial to define its boundary [1]. The deviation from this ‘narrowband’ model becomes more relevant the faster an object moves in relation to the propagation speed *c* and the higher the desired coherent integration gain becomes. One implication is that the achieved resolutions will be increased against stationary objects in case of a high time-bandwidth product. If this is not properly considered, it will however become more uncertain against fast moving objects and the occurring range and Doppler frequency spread will affect the overall detection performance. This will already be the case for a purely linear movement. A resolved Doppler frequency difference within a wide instantaneously processed signal bandwidth may then cause a quite severe bandwidth-dependent impact that it is no longer an extension over some range cells. The principle challenge of Doppler dispersion—which describes the bandwidth-dependent Doppler influence and addresses thereby the range migration—has been studied extensively in the sonar domain in the 1950s and 1960s. It gave the motivation due to a comparably low sound propagation speed $c_{s}⋘c_{\mathrm{em}}$ for integration gains as early as 30dB, see e.g., [2,3]. Even though the electromagnetic wave propagation speed $c=c_{\mathrm{em}}$ is much higher, the same challenge will arise similarly in the case of much faster velocities or in the case of higher time-bandwidth products even for comparable speeds. The task of proper compensation has first been addressed in radar in the SAR domain for ground moving target indication. This application was focused to compensate for cross-range blurring in the Doppler domain with a bandwidth-dependent interpolation approach. Thereby, the Keystone transformation has been introduced to compensate a batched/symbol-wise processing of slow ground moving targets with e.g., up to 14 m/s using a processed sample count around $2^{15}=45\;\mathrm{dB}$. This has been published in 1997 [4] or, respectively, in 1999 [5]. By the same authors, the application to coherent integration has been published [6] in 2007 that is one method for compensation approaches. High coherent integration gain detection is nowadays a particular challenge of passive radar applications. The inherently bistatic configuration typically suffers from low effectively radiated powers towards the upper hemisphere in which common aerial targets are located, comparatively low receive antenna gains and strong direct path interference (DPI) levels directly received from the transmitter(s). This is worse in the case of continuous broadcast emissions with a high instantaneous signal bandwidth $B_{\mathrm{eff}}$. Even with shielding towards the transmitter, the dynamic signal level range can easily exceed 80dB above the averaged reflected target levels. Some exemplary narrowband performance estimations for passive bistatic radar systems are given in [7] et al. Common emitter waveforms are digital broadcasting channels like DAB, DVB-T or DVB-T2 based on orthogonal frequency-division multiplexing (OFDM) with an instantaneous bandwidth up to 7.77MHz at UHF frequencies. More advanced illumination sources include also radar emissions and originally non-classical radar waveforms [8]. The high DPI and stationary clutter levels 20 to 25dB over the noise level are typically addressed by either the signal orthogonality of OFDM [9,10], particular orthogonal MIMO waveforms [11] or e.g., ECA cancellation [12]. Since the actual detection process requires some threshold margin to eliminate false alarms, the common batched range-Doppler implementation [8] has to provide the remaining 60 to 70dB for target detection by coherent pulse compression in its range-Doppler processing.

This publication is focused on the effects that arise particularly for relatively fast moving targets starting with the particular case of a constant effective velocity. It thereby includes a fundamental analysis of a high integration based detection capability that aims to highlight the influence of large instantaneously processed bandwidths. This leads to a better understanding of mostly degradations and their causes in particular if high instantaneous bandwidth waveforms are considered. After an overview about the stationary principle of batched range-Doppler processing in bistatic radars and the relation of the coherent integration gain to the time-bandwidth product are given (Section 2), the limits for not anymore stationary considerations are introduced (Section 2.5). This is followed by examples of the processing with an exceeded limit and a modelling of the Doppler effect in its original scaling relations (Section 3). After this, the implication of mainly a constant effective velocity in a common batched pulse compression implementation is shown (Section 4) and the linear movement impacts are quantified (Section 5). The article proceeds with a discussion of time-scale analysis compensation approaches under practical considerations of target motion and in relation to the results shown (Section 6). Although this analysis tries to be as generic as possible, most effects are given in the context of cyclic prefix OFDM waveforms and findings from DVB-T based passive radar measurement sets.

## 2. Challenges of Pulse Compression with High Time-Bandwidth Products

### 2.1. Stationary Pulse Compression Principle

Modern radars are typically very agile and can often make use of bandwidths which enable resolutions smaller or equal to the target dimensions. By adapting pulse and radiation parameters, different radar scenarios can be addressed individually [13]. In order to quantify the impact of the time-bandwidth product, a definition of these two operands is necessary. This enables a clear differentiation between the covered and effective timespan, respective bandwidths and sampling frequency relations. In this section, the stationary aspects of a range-Doppler analysis shall be recapitulated shortly before the target movement induced effects are introduced. A classical batched range-Doppler processing scheme performs thereby an inherent pulse compression by correlation similar to pulse-Doppler radars [14]. This principle is used to reduce the peak power of short pulses while a similar range resolution capability and covered range can be achieved [13].

**Figure 1:**
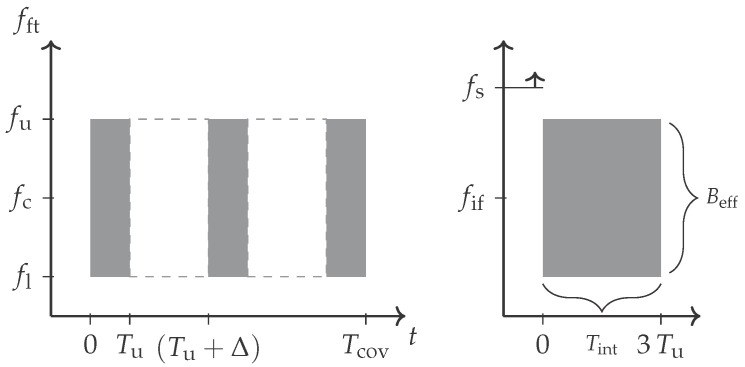
Spectrogram of an emitted pulsed waveform with its *T*-*B* characteristics, RF carrier $f_{c}$, lower $f_{l}$, upper $f_{u}$, baseband intermediate $f_{\mathrm{if}}$ and quadrature sampling frequency $f_{s}$.

During the pulse compression, the received PEP power $P_{\mathrm{rx}}\left(t\right)$ gets integrated within the coherent processing interval (CPI). The actual integration timespan $T_{\mathrm{int}}$ will depend on the waveform and it can be smaller or equal to the CPI duration $T_{\mathrm{cov}}$ as it is shown for a pulsed waveform in Figure 1. This gives a relation of

(1) $$\begin{array}{c} T_{\mathrm{int}}\leq T_{\mathrm{cov}}. \end{array}$$

The received energy $E=\int_{0}^{T_{\mathrm{int}}}P_{\mathrm{rx}}\left(t\right)\;dt$ will hereby be collected over the timespan $T_{\mathrm{int}}$. In the case of additive white noise with a frequency independent noise power per unit bandwidth $N_{0}$, the maximal target return signal-to-noise ratio (SNR) [13,15] will only be worsen by the receiver noise factor *F* to

(2) $$\begin{array}{c} \mathrm{SNR}\leq \frac{E}{N_{0}\;F}. \end{array}$$

In the case of AWGN and low ambiguities, the inequation in (2) becomes maximal. It can however be preprocessed by pre-whitening for coloured noise [15]. Due to the inherent integration stage, actual energy levels are considered (Ws=Joule). A better SNR will obviously lead to an improved detection capability in the presence of noise. If the averaged collected energy is approximated by $E\approx P_{\mathrm{rx},\mathrm{avg}}\;T_{\mathrm{int}}$ and (2) is formulated with noise energy $N\approx N_{0}\;B_{\mathrm{noise}}\;/\;B_{\mathrm{eff}}$, it will result in the fundamental relation

(3) $$\begin{array}{c} \mathrm{SNR}\leq \frac{P_{\mathrm{rx},\mathrm{avg}}}{N_{0}\;F\;B_{\mathrm{noise}}}\;\underset{=\;G_{\mathrm{int}},\mathrm{desired}}{\underset{︸}{T_{\mathrm{int}}\;B_{\mathrm{eff}}}}. \end{array}$$

**Figure 2:**
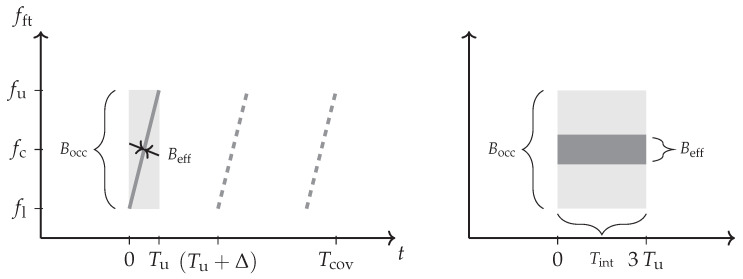
Occupied bandwidth $B_{\mathrm{occ}}\approx f_{u}-f_{l}$ vs. effective instantaneous bandwidth $B_{\mathrm{eff}}$ of an exemplary LFM waveform that is measurable before chirping or after dechirp-on-receive operation.

The involved bandwidth definitions of the waveform may however differ. The occupied bandwidth $B_{\mathrm{occ}}$ will describe the maximum range resolution in a stationary channel. The effective instantaneous bandwidth $B_{\mathrm{eff}}$ contributes to the integration gain against noise and it sets the dynamic range extent below the averaged noise level. The example of a chirped waveform has been illustrated in Figure 2. It has a comparatively low $B_{\mathrm{eff}}$ but it measures a large bandwidth $B_{\mathrm{occ}}=f_{u}-f_{l}$ over the pulse duration $T_{u}$. This relation yields $B_{\mathrm{eff}}\leq B_{\mathrm{occ}}$. Further, the bandwidth dependency in (3) will vanish if $B_{\mathrm{noise}}\approx B_{\mathrm{eff}}$ but only in the case that a stationary channel and target response can be considered. This becomes particularly important in a non-stationary channel with moving targets (Section 2.5).

### 2.2. Batched Processing Scheme Implementation for Range-Doppler Analysis

**Figure 3:**
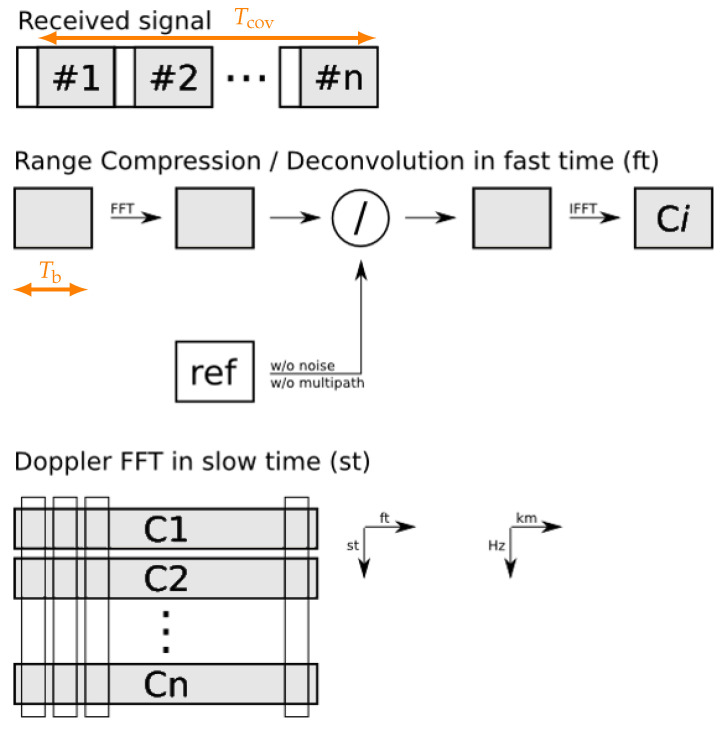
Batched range-Doppler processing principle to build a fast-time (ft) and slow-time (st) domain and to speed-up the radar processing of a CP-OFDM waveform with a block duration $T_{b}=T_{u}$.

The common implementation of the range-Doppler analysis inherits the pulse compression principle. This fundamental batched processing approach has been illustrated in Figure 3 for a cyclic prefix orthogonal frequency-division multiplexing (CP-OFDM) waveform. It uses short blocks with a duration $T_{b}$ that are cut from the received stream (i.e., pulses). Theses blocks are then piecewise correlated with a reference pulse over the fast-time (ft) domain to obtain the measured range $r_{\mathrm{bi}}$. This step is followed by a Fourier transform over *n* processed blocks $c_{i}$ in the slow-time (st) domain. The Doppler frequency $f_{d}$ is thereby evaluated over the timespan $T_{\mathrm{cov}}$ at each individual range bin. Because the pulse compression is performed over the fast-time pulse duration that represents the range, it is also called range compression. This approach will in the case of a CP-OFDM waveform further profit from signal orthogonality but the block duration has to match the symbol duration $T_{b}=T_{u}$ [9,10]. Instead of a direct cross-ambiguity function evaluation [13], this approach performs a short-term correlation over the short blocks plus an additional Doppler frequency analysis. Common software-defined radars will further work on digital represented and discretely sampled bins of the actual received signal. The quadrature sampling rate $f_{s}$ has then to be

(4) $$\begin{array}{c} f_{s}\geq B_{\mathrm{eff}}. \end{array}$$

Obviously, a waveform with a high instantaneous effective bandwidth $B_{\mathrm{eff}}$ will then require a higher quadrature sampling rate $f_{s}$ as expressed in Equation (4). This gives a bandwidth implication that is mainly determined by the implementation architecture and will be important later. This batched approach provides a convenient way for fast time (ft) slow-time (st) domain range-Doppler analysis. The scheme can thereby make use of efficiently implementable fast Fourier transform (FFT) algorithms to achieve a cost function of O(nlogn) for arbitrary *n* [16]. Hereby, also the range compression can be performed in the frequency domain [8] as it has been illustrated in Figure 3. The scheme benefits from a significant increase in processing speed [14,17] and it is similarly used for FMCW pulse compression radar [13] with chirped waveforms—like the one shown in Figure 2—but the evaluated timespan $T_{\mathrm{cov}}$ will be in this case slightly decreased by the ramp duration $T_{u}$. The typical dechirp-on-receive approach also turns out to be a closely approximated matched filter processing [18]. It inherently assumes a piecewise stationary fixed target range and a constant effective velocity over the timespan $T_{\mathrm{cov}}$. The target movement is partly addressed in this processing scheme. An approximation is hereby a constant phase over the block duration $T_{b}$ that is introduced by the analysed Doppler frequency $f_{d}$ [9] and, respectively, a stationary channel within $B_{\mathrm{occ}}$ e.g., during a chirp. If all the approximations are valid to consider, the inherent matched filter design will follow the stationary energy relation of Equation (3). This will be evaluated in the discretised and ambiguously processed range $r_{\mathrm{bi}}$ and Doppler frequency $f_{d}$ domain. Its particular implementation specific movement implications will be discussed in Section 4.

### 2.3. Evolution of Radar Waveforms and Reasons for High Instantaneous Time-Bandwidth Products

In order to cope with different radar measurement tasks, the processed time-bandwidth parameters of the waveform and the modulation design can be pushed. Since it is strongly coupled to waveform design, this part shall not be addressed in detail. In general, large time-bandwidth products come along with high resolution parameters and large possible coherent integration gains. Both can be desired for several reasons. A high range resolution and accuracy can be achieved with a high bandwidth ($B_{\mathrm{occ}}$ +) and it is beneficial for an improved positioning and localisation capability, especially in multistatic configurations. The maximal achievable range resolution in a stationary channel with propagation speed *c* and without parametric super-resolution can in general be approximated by

(5) $$\begin{array}{c} \delta r_{\mathrm{res}}\geq \frac{c}{2\;B_{\mathrm{occ}}}=\frac{c}{2\;(f_{u}-f_{l})}. \end{array}$$

In order to support velocity based tracking and particular aspects of object related classification, a better velocity or micro-Doppler resolution ($T_{\mathrm{cov}}$ +) can be desired. The smallest resolution in the analysed Doppler frequency $\delta f_{d}$ can be estimated [13] directly from the processed CPI duration as

(6) $$\begin{array}{cc} \delta f_{d} & \geq \frac{1}{T_{\mathrm{cov}}}. \end{array}$$

The third ambition can be a large instant dynamic range from the highest to the lowest signal level whereby the waveforms may have a thumbtack or pin head shaped auto-correlation property with a plateau depending on the signal structure [10,13]. The desired coherent integration gain from relation (3) is usually defined as the integration time-effective bandwidth product to

(7) $$\begin{array}{c} G_{\mathrm{int},\mathrm{desired}}=T_{\mathrm{int}}\;B_{\mathrm{eff}}. \end{array}$$

This will enable a processing approximately down to $T_{\mathrm{int}}\;B_{\mathrm{eff}}$ below the noise level. A high instantaneous effective bandwidth can thereby pose particular challenges in the radar signal processing chain with respect to linearity, beamforming, hardware stability and delay realisation. With respect to relatively high desired integration gains, the target movement becomes an increasingly considerable factor (Section 2.5). The processing might then need to consider the Doppler influence in more detail (Section 3) and treat it in its actual scaling relation (Section 6.3). In particular for high instantaneous bandwidth waveforms like OFDM the movement impact can be quite strong and it is often underestimated. This is important in for example a passive radar view of coherent multi-channel systems that include ‘neighbour channels’ in the scenery in their processing. If the increase is high and the Doppler effect is not treated appropriately, an uncompensated coherent range-Doppler processing will lead to the bandwidth paradox (Section 5.4). Despite more spectrally distributed power was available for an increased $B_{\mathrm{eff}}$, an integration would worsen the result if the stationary assumption (Section 2.5) is violated. Similar effects can be observed for increased covered timespans $T_{\mathrm{cov}}$. These particular aspects of pulse compression radar and a closer look on the target returns shall be addressed in the following sections.

### 2.4. Bistatic Geometry Relations on the Effectively Measurable Velocity

**Figure 4:**
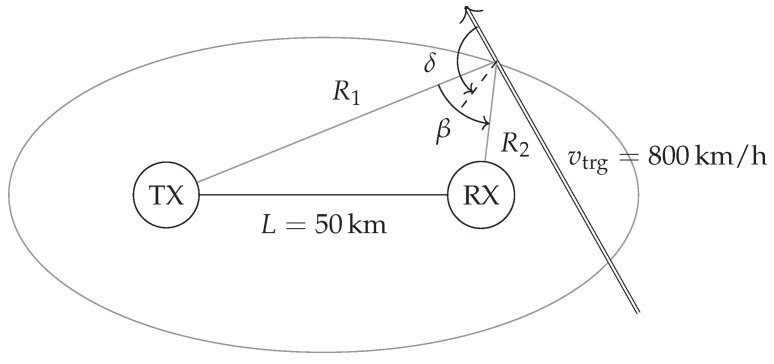
Bistatic setup with a baseline of L=50km between a transmitter (TX) and receiver (RX) pair with bistatic angle β and a target moving in direction δ at a speed of $|{\vec{v}}_{\mathrm{trg}}|=800\;\mathrm{km}/h$ over ground.

An important movement consideration is that the target speed $\left|{\vec{v}}_{\mathrm{trg}}\right|$ will only be partly effective against a single sensor. This has to be considered in for example a typical passive radar (PR) scenario that is at least bistatic because it is based on the illumination by a third party transmitter in the radar scenery. Although most PR systems are multistatic, the fundamental issues can often be broken down to their bistatic relation. This has been illustrated in Figure 4.

The general situation present in a bistatic passive radar is that it inherits a weak illumination of the target, a strong direct path interference (DPI) at the receiver node and challenging waveforms. A large instant dynamic range and high coherent integration gain are therefore required for target detection. The bistatic range towards the target can be defined as $R_{\mathrm{bi}}=\left(R_{1}+R_{2}-L\right)$ whereby the baseline *L* in each transmitter and receiver pair can easily exceed 30km. The effective velocity $v_{\mathrm{eff}}$ that is directly measurable at the receiver node will be smaller or equal to the target speed $\left|{\vec{v}}_{\mathrm{trg}}\right|$. This relates in a bistatic geometry to the bistatic angle β and velocity angle δ relative to β bisector as [19]

(8) $$\begin{array}{c} v_{\mathrm{eff}}=v_{\mathrm{trg}}\;\cos\delta \;\cos\frac{\beta }{2}. \end{array}$$

A time-dependent bistatic velocity $v\left(t\right)=v_{\mathrm{eff}}\left(t\right)$ can hereby include an acceleration of the effective velocity that is geometry-induced by the relative target position along its trajectory or due to an actual target acceleration. Common processing approaches assume a constant effective velocity. This case will be treated in more detail in the following and it will later be related to practical considerations.

### 2.5. Limits Introduced by the Bistatic Velocity and the Time-Bandwidth Product

As it has been pointed out previously, the shown fundamental axioms are based on the assumption of a piecewise stationary channel. This view is obviously limited for moving target detection. Most classical range-Doppler processing schemes are however still based on the piecewise assumption of a single Doppler frequency displacement, a constant effective velocity and a fixed bistatic range. The movement is thereby implicitly treated by just its Gross Doppler frequency displacement $f_{D}$ of the RF carrier $f_{c}$ and at a single time delay. This is equally valid for the common batched implementation scheme of a range-Doppler analysis that has been described in Section 2.2. The pulse compressed energy retrieved by matched filtering will hereby be independent of the processed bandwidth according to Equation (3) as long as the channel with the target return can be considered stationary (Section 2.1). The common and often called ‘narrowband’ boundary for classical range-Doppler processing is generically defined to confine a point scatterer movement to one range cell with propagation speed *c* as [1]

(9) $$\begin{array}{cc} \frac{2\;v_{\mathrm{eff}}}{c} & \ll \frac{1}{T_{\mathrm{cov}}\;B}. \end{array}$$

The bandwidth *B* in Equation (9) has thereby not been clearly defined yet. It can be either the occupied bandwidth $B_{\mathrm{occ}}$ or the quadrature sampling rate $f_{s}\geq B_{\mathrm{eff}}$ (4) at which the sampled data is represented. More precisely expressed, the constant velocity leads to the two following constrains. First, the analysed frequency resolution $\delta f_{d}\approx T_{\mathrm{cov}}^{-1}$ shall be larger than $\frac{2\;v_{\mathrm{eff}}}{c}\;\left(f_{u}-f_{l}\right)$ to confine the Doppler extent. Second, the bistatic range change of $v_{\mathrm{eff}}\;T_{\mathrm{cov}}$ shall be smaller $c\;/\;(2\;f_{s})$ to limit the extent to the range cell spacing $\delta r_{\mathrm{bi}}$ ($\propto f_{s}^{-1}$). If both conditions are combined, this will give an upper boundary on $G_{\mathrm{int}}$. The stationary boundary for a linear movement that is valid for $f_{s}\geq B_{\mathrm{occ}}$ then yields

(10) $$\begin{array}{cc} G_{\mathrm{int}}\leq T_{\mathrm{int}}\;B_{\mathrm{eff}}\leq T_{\mathrm{cov}}\;B_{\mathrm{occ}}\; & \overset{!}{\leq }\;\frac{c}{2\;v_{\mathrm{eff}}}\leq {\mathrm{TB}}_{\max}\approx T_{\mathrm{cov}}\;f_{s}. \end{array}$$

The boundary shown in Equation (10) could be slightly relaxed if the sampling rate is within the range $B_{\mathrm{eff}}\leq f_{s}\leq B_{\mathrm{occ}}$ from Equation (4) for low $B_{\mathrm{eff}}$ since the instantaneous Doppler spread is $\propto B_{\mathrm{eff}}$ and will tend to $\Delta f_{D}$ acc. to Equation (15) on every extended range bin if $B_{\mathrm{eff}}\to B_{\mathrm{occ}}$.

For the next considerations, the wide instantaneous bandwidth of a broadcast OFDM waveform will be considered so that the approximation $B_{\mathrm{occ}}\approx \left(f_{u}-f_{l}\right)\approx B_{\mathrm{eff}}\approx f_{s}$ is fulfilled. The explicit key parameters computed for a fixed time-bandwidth product with the speed of light in vacuum $c=c_{\mathrm{em}}$ and a constant effective velocity of $v_{\mathrm{eff}}=700\;\mathrm{km}/h=194\;m/s$ are shown in Table 1. It contains various sets of timespans and bandwidths with their maximal achievable range resolutions and analysed frequency accuracies for pulse compression. Hereby, all sets have a maximum integration gain from Equation (7)

(11) $$\begin{array}{c} G_{\mathrm{int}}\leq T_{\mathrm{int}}\;B_{\mathrm{eff}}\leq {\mathrm{TB}}_{\max}=58.8\;\mathrm{dB}. \end{array}$$

Related to the values expressed in Table 1, the integration time will roughly be limited to 50ms before range walk occurs if a maximal range resolution down to 10m is achieved. However, the pairs cannot be treated and compared independently of the channel and target response. In particular, if the resolution gets smaller than the target dimension, it will likely introduce bandwidth-dependent and RCS modelling related effects so that each set might perform differently. This effect can even be present within the 8MHz bandwidth of a single DVB-T channel as it will be shown in Section 5.5 for actual target return measurement examples. The coherent processing of a ‘high resolution mode’ has been further discussed in e.g., [11,20]. The consideration of a fixed (resolution based) integration limit from Equation (10) seems at first plausible but it might be required to exceed this limit set on the maximum time-bandwidth product. This can be desired either because not all objects are moving with maximum speed, a finer ground resolution is desired or simply a higher coherent integration gain is needed for the detection of small radar cross sections or to achieve an increased coverage. Thereby it is important that a difference of solely 6dB relates to a factor of $\sqrt{2}$ in covered monostatic range. This difference can even be a factor of up to 2 in one-sided bistatic range because the inequation $R_{1}^{2}\;R_{2}^{2}\leq {\left(R_{1}+R_{2}\right)}^{4}$ is valid in the oval of Cassini power relation [19]. This raises the question of improving this limitation and which effects, loss expectations and further considerations need to be kept in mind for finer resolutions or faster moving objects. If more than 59dB as related to Table 1 shall be achieved in the case of $v_{\mathrm{eff}}=700\;\mathrm{km}/h$, then a loss will have to be considered without a properly compensated processing. This will happen equally if the bandwidth or time is increased or e.g., a processing fixed to 60dB is used.

In particular bistatic passive radar applications often require an even higher coherent integration gain to enable a reliable target detection at bistatic ranges of multiple tenfold kilometres. Since most targets are moving, this boundary becomes highly relevant for effective velocities larger than 200 to 300km/h and desired integration gains that exceed 65dB. If the target changes its bistatic range within the duration of the CPI significantly and this violates Equation (9), the range walk effect will occur but it will then not only cover some range cells for common effective bandwidths $B_{\mathrm{eff}}$ of broadcast emissions (Section 3) although it is primarily considered as range migration in standard radar definitions [21]. An actual extended target can further introduce a distribution over several range cells [11] for a high resolution waveform if the target dimension is larger than the resolved bistatic range cell area ($\propto f_{s}^{-1}$). The main implication is that a point scatterer return will not be fixed to one range cell and further a Doppler spread may occur even for a constant effective velocity so that a target track might be lost. This is shown in the next Section 3 and its impact will be quantified in Section 4 and Section 5.

## 3. Doppler Effect Modelled in Its Scaling Relation

### 3.1. Exemplary Processed Targets from Measurement Campaign Datasets

In the following, target responses from actual passive radar (PR) datasets are shown. The examples were intentionally processed with a ‘too high’ time-bandwidth product that exceeds the boundary defined in Equation (9) to highlight the involved effects. Thereby, an orthogonal frequency-division multiplexing (OFDM) waveform obtained from a single channel DVB-T in the UHF frequency range was used. This digital broadcast emission inherits both aspects of a high desired integration gain with an integration time-effective bandwidth product according to Equation (7). It provides a continuous illumination and it has a high instantaneous effective bandwidth of $B_{\mathrm{eff}}\approx B_{\mathrm{occ}}=7.61\;\mathrm{MHz}$. The waveform also features good auto-correlation properties so that the 8k mode symbol duration $T_{u}=896\;\mu s$ has been used as block duration $T_{b}$ of the batched processing scheme. The illustrated bistatic sampling is at 33m per cell. All intensity levels in the bistatic range-Doppler diagrams are plot on a logarithmic scale.

**Figure 5:**
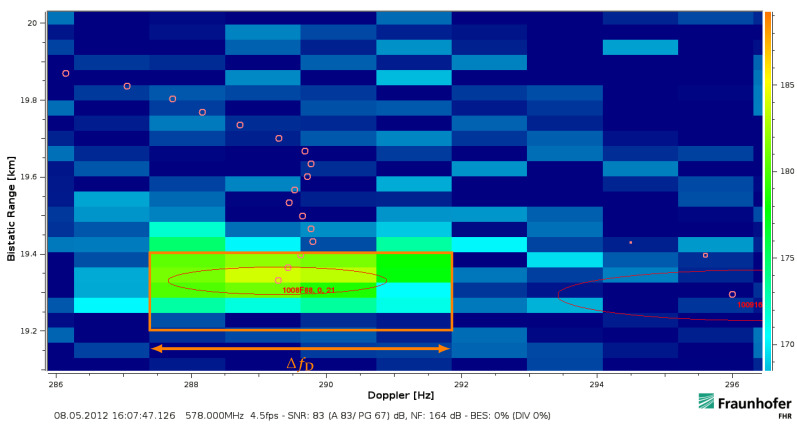
Target tracked with an approximately constant effective bistatic velocity v≈270km/h and $T_{\mathrm{cov}}=896\;\mathrm{ms}$ with $T_{\mathrm{int}}=717\;\mathrm{ms}$, spread fluctuations led to a detached track visible on the right side.

The target return shown in Figure 5 had been accelerated and then moved with an approximately constant bistatic velocity of about 270km/h in the processed interval. It has intentionally been processed with a covered timespan $T_{\mathrm{cov}}$ times effective bandwidth $B_{\mathrm{eff}}$ equivalent to 68dB. Thereby, the return becomes extended by $\Delta f_{D}=4.5\;\mathrm{Hz}$ in Doppler (4 cells) and by 200m in bistatic range (6 cells). The effective velocity corresponds to a bistatic range walk of 134.40m (4 cells) within the covered timespan $T_{\mathrm{cov}}=896\;\mathrm{ms}$. The two additional range cells could fit to a resolved target extension with an assumed length of 30m (≤2 cells) but the extension over $\Delta f_{D}$ is contradicting to its linear movement. The energy becomes spread over 20 cells (13dB), which is much more than just 6(=4+2) bins if a pure extension in range was considered. This ‘rectangular spread’ can be explained by the Doppler frequency difference $\Delta f_{D}$ that is resolved within the signal bandwidth. This particular effect of a linear movement will be analysed in the next Section 3.2. Another implication of the extended and fluctuating target return is well visible in the lower right of Figure 5, another track head detached from the actual target at 19.6km that was likely caused by the sudden motion change and the spread influence.

**Figure 6:**
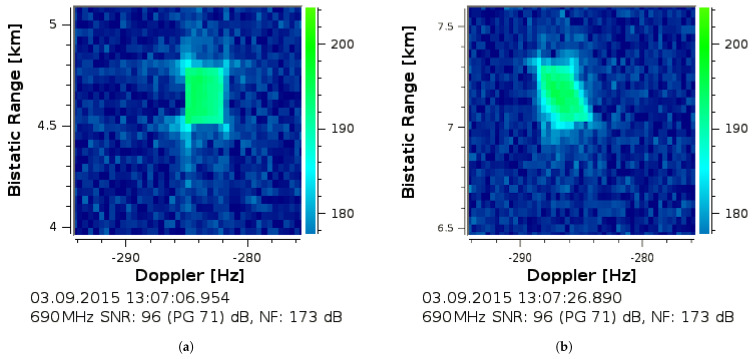
Strong departing target return with a segment of constant effective bistatic velocity and slight acceleration processed with increased $T_{\mathrm{int}}=1.79\;s$ and $T_{\mathrm{cov}}=2.24\;s$ with 1/4 as guard interval fraction. (**a**) Constant bistatic $v_{\mathrm{eff}}\approx -220\;\mathrm{km}/h$. (**b**) Slightly accelerated at $v_{\mathrm{eff}}\approx -225\;\mathrm{km}/h$.

An additional example of a strong return response from a close-by target is shown in Figure 6a. It has departed with a constant bistatic velocity within an even more extended duration of $T_{\mathrm{cov}}=2.24\;s$. Contrary, in Figure 6b a small acceleration is visible at the start of the integration. The target return remained hereby in both cases almost uniform in intensity over the covered $T_{\mathrm{cov}}$ and occupied $B_{\mathrm{occ}}$ at its close bistatic range since it did not fluctuate and it was quite strong not to be overlapped by noise.

### 3.2. Effect of a Constant Effective Velocity on an Instantaneous Broadband Waveform Processing

Matched filter based processing concepts are independent of the signal bandwidth only for stationary considerations as shown in (3) before. Thereby it can be regarded an optimal filter in the presence of preferable white noise [15]. This consideration is limited for moving objects due to the fact that these do not always result in temporary stationary signals. In order to explain the counterintuitive finding of an additional extent over the Doppler frequency and the surface spread, the Doppler effect has to be analysed more in-depth. In a non-stationary situation, it becomes necessary to model the Doppler effect in its original scaling [22,23] relations. This is an actual dilation or compression of a scattered signal [1,3]. It has hereby to be taken into account that the Doppler effect occurs for radar a second time at the scattering point in contrast to an emission from a moving object. Since the classical processing in range-Doppler follows the Fourier relations in the frequency domain, the laws of Fourier transform may be used. This consideration follows the time scaling property of the Fourier transform (FT) so that it is equal to frequency compression or dilation of the received signal x(t),

(12) $$x(st)⊶\frac{1}{\left|s\right|}\;X\left(\frac{f}{s}\right)$$

with a time scale factor *s* expressed for a target velocity v=const [1,23] as

(13) $$\begin{array}{cc} s & ={\left(\sqrt{\frac{c-v}{c+v}}\;\right)}^{2}=\frac{c-v}{c+v}. \end{array}$$

The scaling relation in Equation (13) is then the non-relativistic case for electromagnetic waves with an effective velocity *v* equal to an object observed from its heading that moves with *v* (i.e., cosφ=0 [23]) as long as the actual |vs.|≪c so that just longitudinal Doppler scaling is treated. The scale factor *s* is in the order of $1\pm 10^{-8}$ to $1\pm 1.5\times 10^{-6}$ in case of effective velocities between 5km/h and 800km/h for electromagnetic waves with a propagation speed *c* in vacuum $\left(c=c_{\mathrm{em}}=c_{0}\right)$ or, respectively, air. Due to these small scale factors, the change in peak intensity caused by motion [22], which is considered as 1/|s| in Equation (12), can usually be neglected. The Doppler frequency displacement $f_{D}$ of only a single RF carrier scaled at $f_{c}$ will relate to the narrowband approximation of a single Doppler frequency shift by

(14) $$\begin{array}{cc} f_{D}\left(v,f_{c}\right) & =\left(1-s\right)\;f_{c}\approx \frac{2\;v}{c}\;f_{c}. \end{array}$$

For all sub-carriers $f_{c}\in \left[f_{l},f_{u}\right]$ within $B_{\mathrm{occ}}$, this leads to a Doppler frequency spread

(15) $$\begin{array}{cc} \Delta f_{D} & =\left|s-1\right|\;\left(f_{u}-f_{l}\right)\approx \frac{2\;|v(t)|}{c}\;\underset{B_{\mathrm{occ}}}{\underset{︸}{\left(f_{u}-f_{l}\right)}}. \end{array}$$

**Figure 7:**
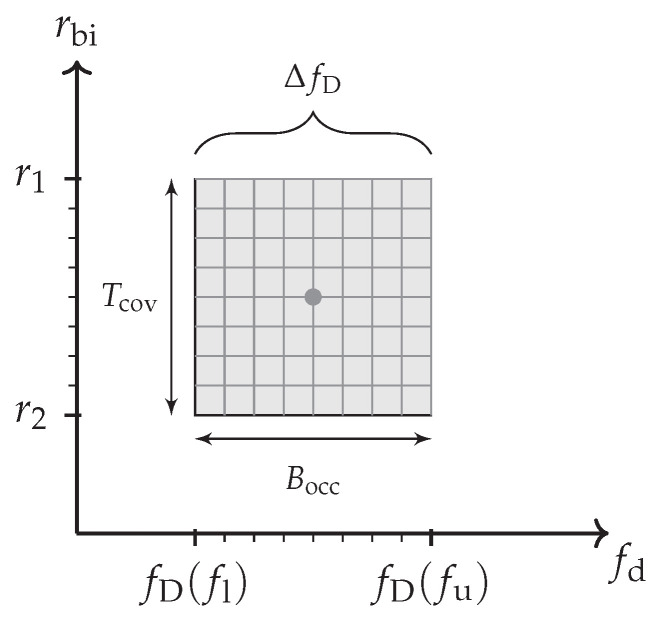
Constantly moving point scatterer extended uniformly in range-Doppler ($B_{\mathrm{eff}}\approx B_{\mathrm{occ}}$).

The bandwidth-dependent scaling relation shown in Equation (15) becomes resolved for a fine analysed frequency resolution $\delta f_{d}$ and a tiny range cell spacing $\delta r_{\mathrm{bi}}$ of the range-Doppler processing. An object that moves linearly from $r_{1}$ to $r_{2}$ will then result in a type of uniform rectangular spread. Particularly, if a broadband waveform—similar to Figure 1—is considered that occupies a wide bandwidth $B_{\mathrm{eff}}\approx B_{\mathrm{occ}}$ instantaneously, the Doppler frequency spread $\Delta f_{D}$ will appear at every passed range cell. This can simply be explained by the fact that the Doppler frequency difference between the lower $f_{l}$ and the upper frequency carrier $f_{u}$ (Figure 1) becomes resolved. Figure 7 contains an illustration of this effect for a point scatterer return that extends in a uniform spread for a high time-bandwidth product.

The covered timespan $T_{\mathrm{cov}}$ will thereby determine the extent in range and the occupied bandwidth the amount of $\Delta f_{D}$. It is noteworthy that this counter-intuitive spread over Doppler frequency is not another effect. According to Equation (12), it is rather an identical consideration of a moving target that changes its bistatic range linearly over the coherent processing interval. Its particular impact (Section 5) on classical range-Doppler processing can become quite severe and is often underestimated. Sometimes this spread is wrongly associated to an assumed change of the effective velocity. The particular impact of this bandwidth-dependent effect on a classical processing scheme will be addressed in Section 5.

### 3.3. Doppler as an Instantaneous Relation

It is important to mention that the Doppler scaling is actually based on the instantaneous effective velocity $v_{\mathrm{eff}}\left(t_{0}\right)=0.5\;d\;R_{\mathrm{bi}}{/dt\;|}_{t=t_{0}}$ that is a differential relation [1] and may not be static. This can be derived from signal phase [13] caused by the bistatic range $R_{\mathrm{bi}}$ change due to a constant velocity to

(16) $$\begin{array}{cc} f_{D}\left(v_{\mathrm{eff}},f_{c},R_{\mathrm{bi}},t_{0}\right) & =\frac{1}{2\pi }\;\frac{\partial \;\Phi (f_{c},t)}{\partial \;t}\;{|}_{t=t_{0}}=-\frac{1}{\lambda (f_{c})}\;\frac{d\;R_{\mathrm{bi}}\left(t\right)}{dt}\;{|}_{t=t_{0}}=\frac{2\;v_{\mathrm{eff}}\left(t_{0}\right)}{c}\;f_{c}. \end{array}$$

It can be seen that the wavelength λ(f) already depends on the frequency *f*. Interestingly, both relations (14) and (16) show this particular frequency dependency over a bandwidth from $f_{l}$ to $f_{u}$ even in the case of a constant effective velocity that is here defined as positive for an approaching target.

## 4. Implication of a Linear Target Movement in Batched Range-Doppler Processing

In the following analysis, the implications on a batched range-Doppler processed target response are illustrated in more detail. It is thereby important to consider the involved influences individually to create a good basis for potential improvements. The first important aspect is that the interpretation of the Doppler effect can be separated for an RF carrier frequency $f_{c}\gg B_{\mathrm{occ}}$. This helps to split the instantaneous Gross Doppler implications mainly related to the processing scheme from to the ones of the bandwidth-dependent part by the movement during the processed interval.

(17) $$\begin{array}{cc} f_{D}\left(f\right) & =\underset{\mathrm{Gross}\;\mathrm{Doppler}}{\underset{︸}{f_{D}\left(f_{c}\right)}}\;+\underset{\mathrm{Bandwidth}\;\mathrm{dependent}}{\underset{︸}{f_{D}\left(f-f_{c}\right)}} \end{array}$$

By Equation (17), the implication of the Doppler effect can be differentiated between physically caused movement impacts and these that are related to the processing scheme.

A batched implementation as shown in Section 2.2 may

**Figure 8:**
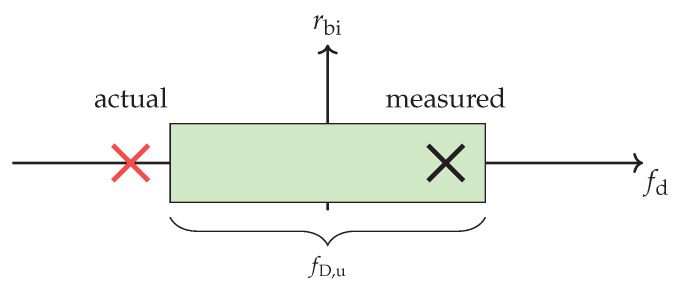
Illustration of the fold-over effect in unambiguous Doppler limited processing.

(a) introduce a frequency sensitivity [14,24] due to the Gross Doppler displacement $f_{D}\left(f_{c}\right)\propto v$. This is caused by its intra-pulse phase approximation [9] over the block duration $T_{b}=T_{u}$. If a cyclic prefix waveform with guard duration α is considered, this will lead to a sensitivity degradation
  (18) $$\begin{array}{c} {\mathrm{Loss}\;}_{\mathrm{PC}}\left(T_{b},f_{D}\left(f_{c}\right),\tau ,\alpha \right)={\left(\frac{1}{\left(1-\frac{u}{T_{b}}\right)\;\mathrm{sinc}\left(\pi \;f_{D}\;\left(T_{b}-u\right)\right)}\right)}^{2}. \end{array}$$
  The relation (18) is thereby only range (τ) dependent with u=max(0,τ−α) outside of the guard duration α [25]. Its derivation is given in Appendix A.
  In addition, only ambiguously measurable parameters are determined by a batched processing. This will impact subsequent processing stages like the tracking. In particular multi-carrier waveforms with long symbol durations $T_{u}$ and a low Doppler tolerance might result in a limited unambiguous Doppler processing from the radar perspective [26]. The unambiguity window in the analysed frequency domain $f_{d}$ is $f_{D,u}=\pm \;0.5/\left(T_{u}+\alpha \right)$ as illustrated in Figure 8. This gives an unambiguously measurable velocity span of $v_{D,u}=\pm \;c/\left(4\;\left(T_{u}+\alpha \right)\;f_{c}\right)$. If the resulting fold-over effect was not considered, it would have a severe impact on tracking because the wrong radial direction and speed would be determined. For chirped signals the ambiguity impact is often worsen by a strong range-Doppler coupling of the waveform [13]. The directly unambiguously measurable range is $R_{\mathrm{unam}}\leq c\;\left(T_{u}+\alpha \right)/2$. This influence can be resolved by pulse staggering or similar modifications to the transmit waveform.

The target displacement in the discretised digital analysis domain of a software defined radar implementation will further cause

**Figure 9:**
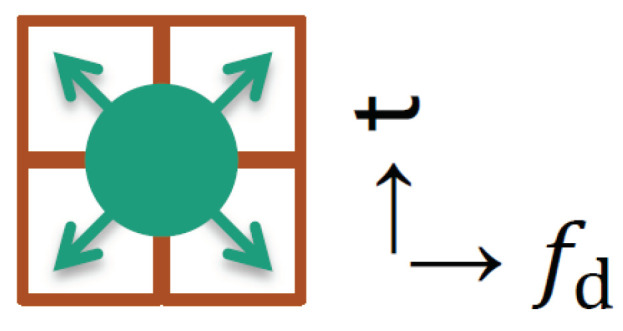
Range-Doppler gate straddling loss of a confined point scatterer return.

(b) a temporary impact on a moving scatterer’s maximum SNR that is inherent from the DSP architecture. This leads to an DFT scalloping [27] and range straddling [13] loss whenever a point scatterer is time-dependently displaced over multiple discretised range (ft) or Doppler (st) cells.
  A single point can be split over up to four processed range-Doppler cells as illustrated in Figure 9. This gets in particular worse for quadrature sampled signals, confined and point scatterer like target returns and rectangular high effective instantaneous bandwidth waveforms. The range straddling impact will increase for high ratios $f_{s}^{-1}\;B_{\mathrm{occ}}$ up to 3.9dB. The DFT scalloping depends on the relation of signal displacement $f_{D}$ to the frequency bins and the number of processed samples. The scalloping loss can reach another 3.9dB for quadrature sampling. Despite this effect is in principle widely known in digital signal processing, it is rarely considered in radar processing schemes [28,29]. This is unrelated to the previously described bandwidth extension spread but the involved challenge might occur for re-focused point scatterer returns as it had been indicated in Figure 7 as well. Particular stationary DSP effects further introduced by sampling, the analogue to digital conversion and deviations from these relations will be addressed in separate publications.

The Doppler moving impact on the bandwidth-dependent $f_{D}\left(f-f_{c}\right),\;f\in \left[f_{l},f_{u}\right]$ will

(c) be determined by the in total occupied bandwidth $B_{\mathrm{occ}}$ and the target motion within the covered timespan $T_{\mathrm{cov}}$. The Doppler’s bandwidth dependent influence relates directly to the target movement but it is at first independent of for example a batched range-Doppler implementation. A high instantaneous bandwidth $B_{\mathrm{eff}}$ will hereby lead to the spread previously illustrated in Figure 7. Due to many distinct impacts, their influence will be addressed in the following section in more detail.

## 5. Influence of Linear Movement within the Signal Bandwidth due to Doppler

This section deals with the particular implication (c) of the Doppler’s bandwidth-dependent contribution as split in Equation (17) in the range-Doppler domain. If a violation of the ‘narrowband’ stationary boundary (Section 2.5) cannot be avoided, certain coherent processing degradations will be a direct consequence of relatively fast and range migrating targets. The arising spread will cause a strong decorrelation and it restrains the maximal achievable integration gain $G_{\mathrm{int}}$ by pulse compression for a range-Doppler analysis. Further, the impact of linear motion on the achievable resolutions (Section 5.2), the decrease of the coherent integration gain (Section 5.3) and arising limitations to multi-frequency channel processing (Section 5.4) will be quantified. Interestingly, the appearing effect can even resolve the response coherency of moving target returns if it is wrongly processed by a classical range-Doppler analysis (Section 5.5). The principal implication of non-linear motion will also be shown (Section 5.6). All identified impacts have to be considered for the development of appropriate compensation and adaptive processing approaches that will be discussed in Section 6.

### 5.1. Spread over Multiple Range-Doppler Cells

Every point scatterer that moves with an effective velocity $v_{\mathrm{eff}}$ will extend in a range-Doppler analysis in range and frequency. This will become visible if the motion is strong compared to tiny achieved processing resolution as it had been described in Section 3.2. If it is considered that the actual range cell spacing depends on the sampling rate $f_{s}$ and the totally covered timespan $T_{\mathrm{cov}}\geq T_{\mathrm{int}}$, the following amount of cells will be resolved with the resolutions earlier expressed in Equations (5) and (6).

(19) $$\begin{array}{cc} \; & \Delta \;\mathrm{rangecells}={\#}_{r}=\left\lceil \frac{v_{\mathrm{eff}}\;T_{\mathrm{cov}}}{c\;/\;(2\;f_{s})}\right\rceil \end{array}$$

(20) $$\begin{array}{cc} \; & \Delta \;\mathrm{dopplercells}={\#}_{d}=\left\lceil \frac{2\;v_{\mathrm{eff}}\;B_{\mathrm{eff}}\;T_{\mathrm{cov}}}{c}\right\rceil \end{array}$$

The total number of affected range-Doppler cells is then the product of (19) and (20) so that it results in a significant surface spread in the case of a large effective instantaneous bandwidth $B_{\mathrm{eff}}$. This yields

(21) $$\begin{array}{cc} {\mathrm{Loss}\;}_{\mathrm{rw}} & =\left\lceil {\#}_{r}\;{\#}_{d}\right\rceil \approx 4\;v_{\mathrm{eff}}^{2}\;T_{\mathrm{cov}}^{2}\;f_{s}\;B_{\mathrm{eff}}/c^{2}. \end{array}$$

### 5.2. Analysis of Decreased Range-Frequency Accuracy

The stationary boundary (Section 2.5) can equally be related to ${\mathrm{Loss}\;}_{\mathrm{rw}}$. It becomes >1 in consequence of an exceedance so that a point scatterer spreads over multiple cells in the range-Doppler domain. The range resolution previously expressed in Equation (5) will be worsen by Equation (19) after uncompensated processing to

(22) $$\begin{array}{cc} \delta r_{\mathrm{res}}\left(B_{\mathrm{occ}},\ldots \right) & \approx \underset{\mathrm{Bandwidth}\;\mathrm{given}}{\underset{︸}{\frac{c}{2\;B_{\mathrm{occ}}}}}\;\underset{\mathrm{Point}\;\mathrm{widening}}{\underset{︸}{\left\lceil {\#}_{r}\left(v_{\mathrm{eff}},f_{s},T_{\mathrm{cov}},\ldots \right)\right\rceil }}. \end{array}$$

Thereby, the resolution of two adjacent scatterers will be decreased due to the spread. Similarly, the achievable Doppler frequency resolution from Equation (6) $\delta f_{d}$ can be expressed for a constant $v_{\mathrm{eff}}$ by Equation (20) as

(23) $$\begin{array}{cc} \delta f_{d}\left(T_{\mathrm{cov}},\ldots \right) & \geq \underset{\mathrm{Time}\;\mathrm{given}}{\underset{︸}{\frac{1}{T_{\mathrm{cov}}}}}\;\underset{\mathrm{Point}\;\mathrm{widening}}{\underset{︸}{\left\lceil {\#}_{d}\left(v_{\mathrm{eff}},B_{\mathrm{eff}},T_{\mathrm{cov}},\ldots \right)\right\rceil }}. \end{array}$$

The widening due to the movement will pose a challenge even for often not strongly extended targets if these are still detectable above the noise threshold. This affects amongst others the positioning capability that multistatic systems often perform by the intersection of multiple bistatic ellipsoids. A measured example—as given in Figure 6—indicates the problem. It becomes more difficult to determine the accurate velocity and range at a common reference time within $T_{\mathrm{cov}}$ from the range-Doppler plot. Even if guessing a ‘rectangular response’ was possible, a blurred return might be difficult to fit to a particular range and velocity. The worse case of a target return that fluctuates strongly within the CPI and over bandwidth will be shown in Section 5.5. Thereby, the measurable accuracy of range and velocity becomes severely affected so that a centre of gravity analysis will not resolve the accurate values a posteriori. Moreover, an uncompensated widening will be challenging for CA-CFAR detection based algorithms because these would need to incorporate the broadening for increased velocity spans. Otherwise, the actual target detection may be missed if the extension around an assumed confined target was mistreated as an increased clutter level. A modification of the CA-CFAR would therefore be required to avoid the spread return in high effective velocity ranges being dropped.

### 5.3. Impact of a Constant Speed on the Achievable Coherent Integration Gain $G_{int}$

Another direct consequence of the spread over multiple range-Doppler cells is that the energy of single confined and point-like scattering points becomes equally distributed over a surface. The target return will start to decorrelate. A kind of integration loss is therefore already given by Equation (21)

$$\begin{array}{cc} {\mathrm{Loss}\;}_{\mathrm{rw}} & =\left\lceil {\#}_{r}\;{\#}_{d}\right\rceil \approx 4\;v_{\mathrm{eff}}^{2}\;T_{\mathrm{cov}}^{2}\;f_{s}\;B_{\mathrm{eff}}/c^{2}. \end{array}$$

Because this range walk related spread depends only on the effective bandwidth $B_{\mathrm{eff}}$, the sampling rate $f_{s}$, the effectively measurable velocity $v_{\mathrm{eff}}$ and the covered timespan $T_{\mathrm{cov}}$, this impact will solely relate to the bandwidth-dependent part of Equation (17) independently of the actual RF carrier frequency $f_{c}$. The stationary and linearly uncorrelated noise components are still suppressed by the matched filter gain but the decorrelation leads to a decreased target level—in presence of the stationary clutter and remaining noise levels. In the special case of a high instantaneous effective bandwidth $B_{\mathrm{eff}}$ and a long covered timespan $T_{\mathrm{cov}}$, this extension as it had already been illustrated in Figure 7 can severely degrade the effective coherent integration gain even in the case of a constant effective velocity $v_{\mathrm{eff}}$.

**Figure 10:**
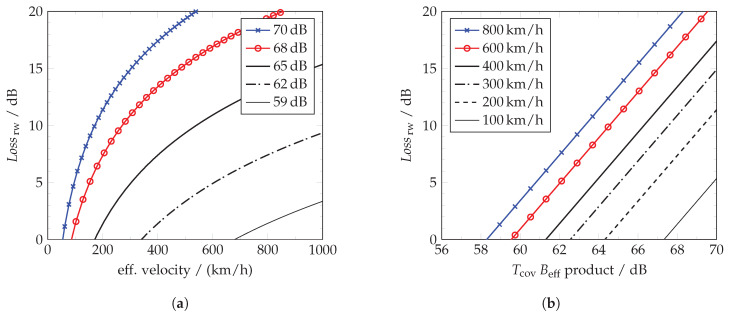
Extension loss in range-Doppler for multiple time-bandwidth products and linear velocities. (**a**) Expressed with $f_{s}\approx B_{\mathrm{eff}}$ for multiple $T_{\mathrm{cov}}\;B_{\mathrm{eff}}$ sets. (**b**) Expressed for constant effective velocities.

Whenever ${\mathrm{Loss}\;}_{\mathrm{rw}}$ becomes larger 1, high time-bandwidth products $T_{\mathrm{cov}}\;B_{\mathrm{eff}}$ cause large integration losses according to Equation (21) in classical range-Doppler processing. This is illustrated for exemplary but typical passive radar parameters in Figure 10a,b. Considering the previous example of $v_{\mathrm{eff}}=700\;\mathrm{km}/h$ in Section 2.5, a desired gain of $T_{\mathrm{cov}}\;B_{\mathrm{eff}}=65\;\mathrm{dB}$ is far too high for classical range-Doppler processing. An introduced loss of 12dB would give a detectable ‘narrowband’ RCS that is by Δ6dB worse than if 59dB had been used and the maximal effective $G_{\mathrm{int}}$ would reduce to 53dB. This is expressed under the consideration that $T_{\mathrm{int}}\leq T_{\mathrm{cov}}$ and an approximated $f_{s}\approx B_{\mathrm{eff}}$. Equally, the minimal detectable RCS would increase from e.g., $0\;{\mathrm{dBm}}^{2}$ to over $+12\;{\mathrm{dBm}}^{2}$ or, respectively, the maximum covered range would decrease in this effective velocity span. Even in the case of $v_{\mathrm{eff}}=300\;\mathrm{km}/h$ a loss of already 5dB would be present. Multiple objects could therefore be missed if just this effect is not considered. It is important that this consideration does not yet include any performance degradation like the Gross Doppler frequency sensitivity of a batched implemented scheme (Section 4).

### 5.4. Bandwidth Paradox in Multi-Channel Processing with a High Instantaneous Bandwidth

A similar but an additionally to be highlighted aspect is that the loss is worsen quadratically by every further increase of the integration time or bandwidth. If a constant effective velocity is considered, the loss added will be magnified by 6dB for every doubling of the time-bandwidth product as illustrated in double log scale in Figure 10b. This will lead to the bandwidth paradox that shall be described in the following. Imagine two coherent transmit channels with the same bandwidth and equal spectral power density being located next to each other. Both channels shall be processed coherently ($B_{\mathrm{eff}}\;+$). Despite the matched filter gain is typically assumed to be independent of bandwidth (Section 2.1) and thereby the combination contains more spectrally distributed energy, it will show a negative dependence in this multi-channel configuration for a violated stationary boundary. If the coherently covered bandwidth is simply extended to process both channels so that $B_{\mathrm{eff}}$ and $f_{s}$ are at least doubled, a loss of 6dB will occur. The effective gain will then be −3dB instead of a gain increased by 3dB. Thus, $G_{\mathrm{int}}$ will not be doubled but halved or, respectively, the detectable RCS will not be doubled but halved if no further compensation is performed. It should hereby be mentioned that for twice the bandwidth, the loss will at least double because $f_{s}\geq B_{\mathrm{eff}}$ as given in Equation (4). Solely if the same $f_{s}$ was used, the desired gain increase would vanish but the resolution would still be lower (Section 5.2).

### 5.5. Target Response Coherency over $T_{\mathrm{cov}}$ and $B_{\mathrm{occ}}$ from Measurement Datasets

An actual extended target return might also differ [11] from a pure uniform return of one dominant confined scatterer as it was the case in the previous examples in Figure 6a,b. While distributed multipath delayed trails behind the target have been observed [30], an extended return can vary as well within the occupied bandwidth over the CPI duration. In the case of range walk and a high instantaneous bandwidth, the target returns become resolved in the range-Doppler plot. This enables a detailed analysis within the covered timespan $T_{\mathrm{cov}}$ and over the whole instantaneously occupied bandwidth.

**Figure 11:**
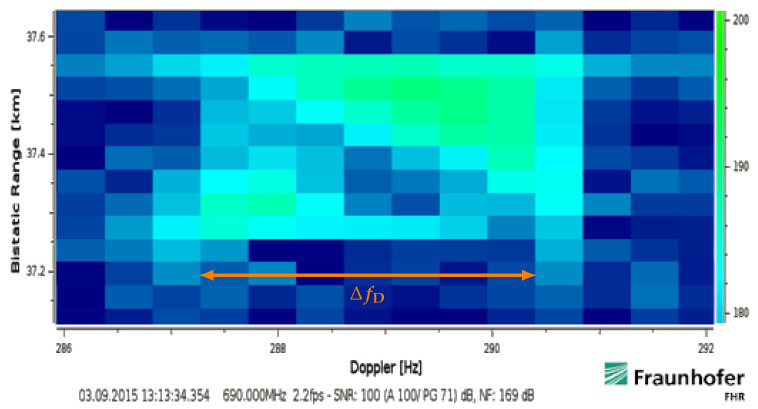
Fluctuating approaching target return moving with a constant v≈225km/h and integrated in $B_{\mathrm{eff}}=7.61\;\mathrm{MHz}$ over $T_{\mathrm{int}}=1.79\;s$ during a coherent processing interval of $T_{\mathrm{cov}}=2.24\;s$.

**Figure 12:**
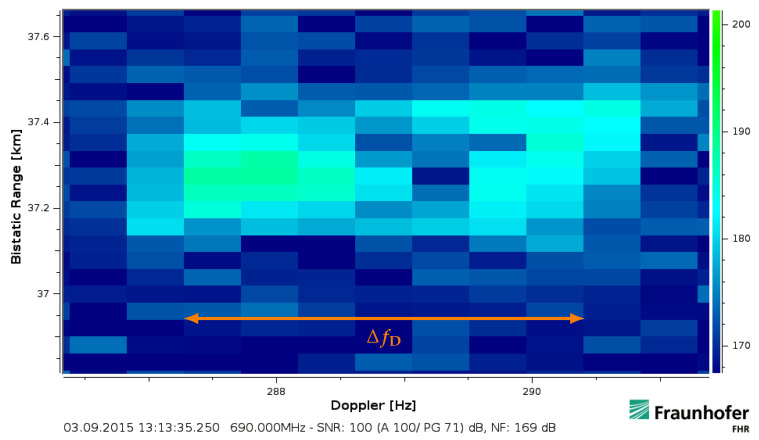
Fluctuating return of the previously shown approaching target with v≈225km/h, shortly after Figure 11 with a deviated target return in $B_{\mathrm{eff}}=7.61\;\mathrm{MHz}$ over $T_{\mathrm{int}}=1.79\;s$ and $T_{\mathrm{cov}}=2.24\;s$.

In order to illustrate these target response fluctuations, a strong target return has been processed with fine resolution parameters of $T_{\mathrm{cov}}\;B_{\mathrm{eff}}=72\;\mathrm{dB}$. It was evaluated at $f_{c}=690\;\mathrm{MHz}$ within $B_{\mathrm{occ}}\approx B_{\mathrm{eff}}=7.61\;\mathrm{MHz}$. The target approached with an almost constant effective velocity during the processed intervals. The result is shown in Figure 11 and the overlapped response 896μs later is shown in Figure 12. It thereby clearly deviates from the uniform spreads of target returns that were shown earlier.

By using Equations (14) and (17), the Doppler frequency difference $\Delta f_{D}=f_{D}\left(f_{u}\right)-f_{D}\left(f_{l}\right)$ can shown to be

(24) $$\begin{array}{cc} f_{D}\left(f_{u}=f_{c}+B_{\mathrm{occ}}/2\right) & =f_{D}\left(f_{c}\right)\;+f_{D}\left(B_{\mathrm{occ}}/2\right) \end{array}$$

(25) $$\begin{array}{cc} f_{D}\left(f_{l}=f_{c}-B_{\mathrm{occ}}/2\right) & =\underset{\mathrm{Gross}\;f_{D}}{\underset{︸}{f_{D}\left(f_{c}\right)}}\underset{\mathrm{independent}\;\mathrm{of}\;f_{c}}{\underset{︸}{-\;f_{D}\left(B_{\mathrm{occ}}/2\right)}}. \end{array}$$

The bar marked with $\Delta f_{D}$ in Figure 11 and Figure 12 shows the return resolved within the CPI duration $T_{\mathrm{cov}}$ from the lower to the upper bandwidth in Doppler frequency over slow-time in steps of

(26) $$\begin{array}{cc} \delta f_{\mathrm{step}} & =c\;/\;(2\;v_{\mathrm{eff}}\;T_{\mathrm{cov}})=1.1\;\mathrm{MHz}. \end{array}$$

Thus, in the case $B_{\mathrm{eff}}\approx B_{\mathrm{occ}}$, this resolves the target return instantaneously at $f_{c}$ within the full spectrum of the occupied bandwidth $B_{\mathrm{occ}}$ over fast-time in a time spacing of

(27) $$\begin{array}{cc} \delta t_{\mathrm{step}} & =\;c\;/\;(2\;v_{\mathrm{eff}}\;f_{s}\;)\;=263\;\mathrm{ms}. \end{array}$$

It can hereby be seen that the target response during its approach with a constant effective velocity might vary strongly over time and even within 8MHz over the bandwidth. If the described aspect is ignored, this will lead to a severe impact on classical schemes. Due to missing parts compared to a purely uniform rectangular response (Figure 6a), this would lead to a specific uncertainty for the posteriori determination of position and speed in an uncompensated range-Doppler processing scheme (Section 5.2). The resolved target fluctuations—likely caused by multipath and aspect angle changes [11,20] or an altered effective antenna gain towards the target—highlight that it is not always optimal to re-focus the whole response within an assumed rectangular spread. The fluctuations could be interpreted similar to ‘narrowband’ Swerling cases [13] if the whole frequency dependent return was simply summed up. However, their original statistical modelling usually follows a slightly different approach.

### 5.6. Higher-Order Motion Aspects

**Figure 13:**
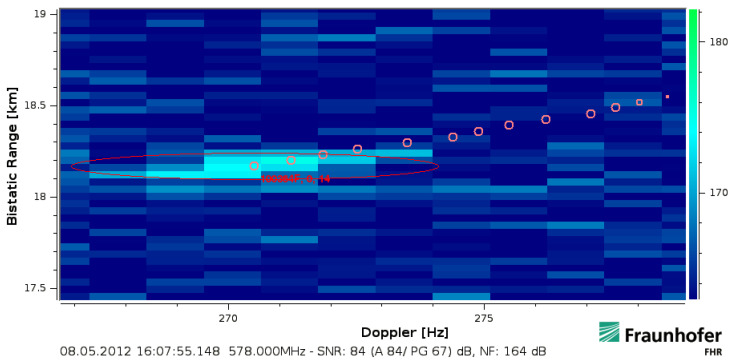
Moderate deceleration in addition to the Doppler frequency spread of a bistatic velocity of $v_{\mathrm{eff}}\approx 270\;\mathrm{km}/h$ with track history indicating the change using $T_{\mathrm{int}}=717\;\mathrm{ms}$ and $T_{\mathrm{cov}}=896\;\mathrm{ms}$.

**Figure 14:**
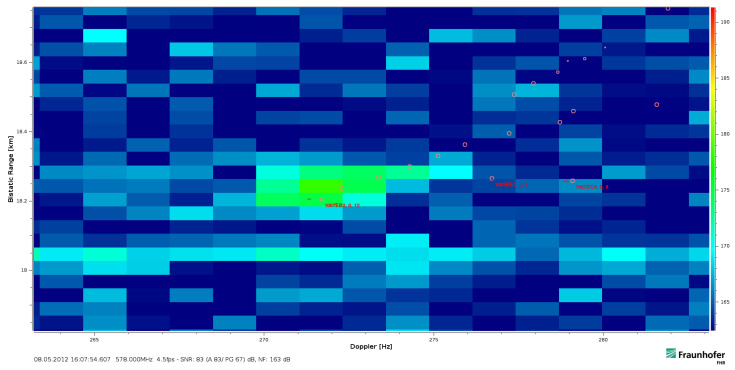
Target previously shown in Figure 5 tracked with a bistatic velocity $v_{\mathrm{eff}}\approx 270\;\mathrm{km}/h$ over $T_{\mathrm{cov}}=896\;\mathrm{ms}$ with $T_{\mathrm{int}}=717\;\mathrm{ms}$, decelerated and noise overlapped that gave multiple track heads.

The actual target motion might further not be strictly linear so that the bistatic velocity changes within the covered timespan $T_{\mathrm{cov}}$ (Section 2.4). In this case, the influence of higher-order motion aspects has to be considered additionally [31]. Hereby, effective acceleration ($a\left(t\right)=d\;v_{\mathrm{eff}}\;/\;d\;t$) and jerk influences could be caused by a complex motion or by the relative position change with respect to the geometry (Section 6.4). In the case of involved motion, a trace as shown in Figure 13 is likely—this is a strong deviation from a rectangular shape. It further extends over the analysed Doppler frequency $f_{d}$ by a deceleration of its effective velocity within the covered timespan. In this example, a simple linear velocity based compensation is certainly counter-productive since it would be based on the rectangular shape in the range-Doppler domain (Section 3). A worse example obtained from quite a weak, spread and decelerated return is shown in Figure 14. Probably due to fluctuations and noise overlap, four track heads were generated by an uncompensated processing so that the track was frequently lost. The contributions of acceleration might be important and considerable beside the velocity-induced implications. Every non-predictable contribution will however open another search dimension in addition to bistatic range and effective velocity for search processing. This will certainly restrain practical implementations to previously covered or presumed target tracks.

## 6. Discussion of Findings and Consideration for Processing Approaches

### 6.1. Preface

The preceding analysis of a common batched range-Doppler processing indicates that the target movement can severely affect its capability to detect and to track relatively fast moving targets. The velocity spans in which linear movement effects need to be specially regarded are these in which the limits of classical ‘narrowband processing’ like the stationary assumption (Section 2.5/Section 5.2) or the first unambiguous Doppler span determined by the processing scheme (Section 4) would be exceeded. Thereby, a common range-Doppler processing scheme is limited to a certain fixed time-bandwidth product for a maximal measured effective velocity (Section 2.5) or, respectively, it is constrained to an acceptable implication at a certain speed (Section 4). This is particularly problematic if a wide velocity span has to be covered. If the introduced implications are not considered, a large coherent integration gain will not be maintained by range-Doppler processing against relatively fast moving target returns. The further analysis indicated that in particular the instantaneous bandwidth-dependent impact (Section 5) and each key parameter related to the movement like the effective velocity gives a strong and quadratic implication for any further increase in case of an already exceeded boundary (Section 5.1). Even counterintuitive relations can then be present so that more loss might be generated compared to the case of no increase (Section 5.4). This is especially important if large desired integration gains were the motivation for a high time-bandwidth product in the first place. In the case that the spread target return was wrongly treated as a confined point scatterer, this could lead in consequence to probably even two close-by targets detected (Figure 12) or several detached track heads as it was visible in Figure 5 and Figure 14. Another necessary condition for coherent processing are actual hardware demands that were not further discussed in this article but are important to be considered as well. Long covered timespans and high instantaneous bandwidths lead to increased demands regarding stability, linearity and intermodulation requirements that have to be properly addressed. Furthermore, the synchronisation to the received signal stream [32] will influence the accuracy and capability of target ranging.

### 6.2. Constant Effective Velocity Detection in a Time-Scale Analysis Approach

A first step to address the linear movement for high time-bandwidth products is to treat the Doppler effect in its original scaling relations even in the case of a constant effective velocity (Section 3). This can be broken down to the question how to properly treat the fine resolutions compared to the Doppler scaling relations for moving target detection. The task becomes ‘how to find the correct scale to treat and to determine the velocity correctly’. The optimal processing technique would thereby address the whole movement effects with a proper method, in short processing time to reduce the added latency and embody a sufficiently low computational complexity and implementation effort. One straight forward approach is a time (range)-scale analysis [1] that results in a modified version of the cross-ambiguity function $\chi (\tau ,f_{d})$ [13]. The modified function with scaling relations then yields

(28) $$\begin{array}{cc} \chi_{\mathrm{mod}}\left(\tau_{0},s\right) & =\int_{t_{0}}^{t_{0}+T_{\mathrm{cov}}}y_{\mathrm{rx}}\left(t\right)\;\frac{1}{\left|x_{\mathrm{ref}}\right|}\;x_{\mathrm{ref}}^{*}\left(\frac{t-\tau_{0}}{s}\right)d\;t\;\;\mathrm{for}\;\left\{s=\mathrm{const}\;|\;t_{0}\leq t<t_{0}+T_{\mathrm{cov}}\right\}. \end{array}$$

The time-scale approach as described in Equation (28) addresses primarily the decreased accuracy due to the involved spread (Section 5.1). Hereby, $t_{0}$ marks the beginning of the CPI with duration $T_{\mathrm{cov}}$ and the time offset $\tau_{0}=R_{\mathrm{bi}}\left(t_{0}\right)/c$ relates to the bistatic target range at a fixed reference point $t_{0}$ in time. The analysed scale factor *s* gives then according to (13) the estimated effective target velocity by $v_{\mathrm{est}}\approx c\;\left(1-s\right)/\left(1+s\right)$. In addition to a regained resolution for linearly moving targets, this also improves the capability for an accurate multistatic localisation because the bistatic range $R_{\mathrm{bi}}\left(t_{0}\right)$ at a common reference time $t_{0}$ will no longer be worsen by the linear movement extension. This approach is in line with the case described in Section 3.2 and the inherent processing model is therefore a continuation of the classical assumption (Section 2.2) of a constant effective velocity $v_{\mathrm{eff}}$ and confined scatterers. It can thereby achieve an increase in the realised coherent integration gain as long as the otherwise extended target return stays similar to a uniform and rectangular spread without deviation (Section 6.4).

### 6.3. Common Time-Scale Analysis Implementation Techniques for Batched Schemes

The main implication of the time-scale approach expressed in Equation (28) is that its implementation requires a modification of the common time-frequency batched processing scheme (Section 2.2). Thereby, the idea is to keep the principle of a batched processing scheme but to compensate the range migration induced spread. The principle implementation methods can be split into two classes depending on their view on the Doppler relation. This is similar to the identical consideration of the scaling in range or in the Doppler extent over the occupied bandwidth $B_{\mathrm{occ}}$ (Section 3.2). The one class tries thereby to address the Doppler scaling relation with a time-domain based compensation by re-scaling, shifting or resampling. The other class performs a normalisation in the frequency domain to compensate the relative Doppler frequency deviation $\Delta f_{D}$ in relation to a reference carrier $f_{c}$. After this compensation has been performed, the radar processing chain can further process the received signal with classical steps.

The first implementation class with a scaling relation in the time-domain can be understood as time re-scaling of the whole received signal. The common effective aircraft velocities between 5km/h and 800km/h cause scale factors of $1\pm 10^{-8}$ to $1\pm 1.5\times 10^{-6}$ in radar (Section 3.2). Several methods are possible whereby the most straight forward approach is rational L/M resampling [1]. Hereby, it turns out that in particular the small factors briefly deviating from 1 are most challenging because these may require large *L* and *M*. Other scaling techniques can be based on different kinds of linear or e.g., polyphase interpolation signal scaling [33] or multi-rate signal processing. Another method is to use a precomputed filter based on a pre-known reference signal [34]. The fractional delays form hereby another set of time-based methods that introduce small time delays before individual compressed pulses to address a particular target motion [35], the actual shift is however implementable in the frequency domain as well. These time-domain re-scaling methods have to be performed either target specific whereby a particular target motion could be addressed or alternatively for multiple velocity spans. Hereby, a limited set of spans is needed because a small deviation $|\Delta \;v_{\mathrm{eff}}|<100\;\mathrm{km}/h$ leads to a difference of only a fraction of one to two dB according to Figure 10b. However, this condition tightens for higher time-bandwidth products so that smaller spans had to be used.

Alternatively, a bandwidth-dependent view as expressed in Equation (15) forms the second class. The idea is to perform a normalisation of the Doppler frequency deviation $\Delta f_{D}$ within the occupied bandwidth by relating the fast-time carriers—that are subject to slightly different Doppler shifts—to that of a fixed reference carrier $f_{c}$. A common technique for high instantaneously processed bandwidths is the Keystone transform (KT) [36,37]. It is based on the ft/st domain of a batched processing scheme (Section 2.2). The KT compensation works as originally described to rescale the slow-time axis $t_{\mathrm{st}}$ of every active fast-time frequency sub-carrier $f_{\mathrm{ft}}\in \left[f_{l},f_{u}\right]$ within $B_{\mathrm{occ}}$ in the slow-time domain by transforming [5,6]

(29) $$\begin{array}{c} t_{\mathrm{st}}\left(f_{\mathrm{ft}}\right)=\left(\frac{f_{c}}{f_{\mathrm{ft}}+f_{c}}\right)\;t_{\mathrm{ks}}. \end{array}$$

The advantage of the Keystone transform is that the whole unambiguous range-Doppler window is compensated for its linear velocity migration [38,39]. It is hereby almost too accurate because it addresses all resolved velocities in the unambiguous Doppler window. The implementation has to consider that this technique requires an interpolation of every fast-time sub-carrier so that the effort increases proportional to this count. A DVB-T2 waveform can for example incorporate up to 27,841 active sub-carriers in one 32k OFDM symbol duration of $T_{u}=3.5\;\mathrm{ms}$ in 8MHz channels [25,40]. Depending on the interpolation method, this can lead to a significant amount of added latency. In order to speed up the computation, several publications focused on the implementation of efficient interpolation methods to reduce the added complexity and the introduced latency [41,42,43]. The processing effort could also be decreased by skipping e.g., every second fast-time sub-carriers to reduce the total number of carriers. Hereby their energy would be lost and the unambiguous range—which is in this case typically not a problem—is halved but the constant velocity spread effect is still properly addressed. Since this transform is commonly applied after pulse compression on the ft/st pulse compressed matrix [41,42], an unambiguous Doppler limited processing has to identify the relevant unambiguous range-Doppler window separately. This is typically required if the block duration $T_{b}$ is determined by a given waveform (Section 2.2) that leads to the fold-over effect in Doppler as it has been illustrated in Figure 8. One method to address the next unambiguous window after Equation (29) is to multiply the term $\exp(-j\;2\pi \;\mathrm{foldover}\;\left(f_{c}/\left(f_{\mathrm{ft}}+f_{c}\right)\;m\right)$ to the st-domain [36,44]. For this, the foldover or ambiguity number [6,36] ≠0 needs a heuristic approach for likely values or a proper identification method like [26]. This is in general necessary to obtain the correct velocity estimate from a detection and to establish a target track. Further, the Gross Doppler sensitivity loss of a batched implementation (Section 4) will not be addressed by this multiplication if the pulses had already been compressed.

Another despite similar method of this normalisation class is to compensate the KT based frequency relation of Equation (29) in the time domain as a dispersive time-domain correction. This tries to address the problem over all the sub-carriers in a dispersive relation over the ft-bandwidth $B_{\mathrm{occ}}$ that makes it possible to implement this compensation in the time-domain as a dispersive FIR filter. It can then be moved to arbitrary positions in the processing chain and therefore also before the pulse compression. This method has been described in 2019 as time-domain keystone transform in [45]. Since different kinds of dispersive implementations are possible for speed-up, this way is promising to address the bandwidth-dependent Doppler implication (Section 5) by this time-scale approach.

Solely in the case of a narrowband waveform, it is a valid technique to multiply an extra single phasor by a complex exponential function to correct a narrowband chirp related to the Stolt interpolation [46]. This can address the bandwidth influence over the occupied bandwidth $B_{\mathrm{occ}}$ as it is used in SAR processing to address the relative platform motion and it is easier but it will only work for an instantaneous narrowband chirped waveform with a low effective bandwidth $B_{\mathrm{eff}}$ – as shown in Figure 2—because it may address the bandwidth-dependent Doppler during the chirp duration $T_{u}$ sequentially. The extra phasor can be related by its range profile [18]. It works similar to a single carrier signal $x_{\mathrm{sc}}\left(t\right)=e^{-j2\pi \;f_{c}\left(t\right)\;t}$ scaled by Equation (12) to $x_{\mathrm{sc}}\left(s\;t\right)=1/\left|s\right|\;\left[\;x_{\mathrm{sc}}\left(t\right)\;\right]\;e^{-j\;2\pi \;f_{D}\left(f_{c}\right)\;t}$ so that it is displaced by the Fourier shift theorem due to its piecewise Doppler frequency

(30) $$\begin{array}{c} f_{D}\left(f_{c}\right){|}_{f_{c}=f_{c}\left(t\right)}\approx \left(1-s\right)\;f_{c}\left(t\right) \end{array}$$

which is approximately $f_{D}\left(t\right)\approx f_{c}\left(t\right)\;\left(2\;v_{\mathrm{eff}}\right)/c$ from Equation (14). This method can however not be applied to compensate range migration of processed high instantaneous time-bandwidth product waveforms.

### 6.4. Constrains of Actual Non-Uniform and Non-Rectangular Target Returns

A time-scale analysis approach with an inherent constant scale factor *s* (Section 6.2) considers a pure uniform rectangular return spread, regardless of the chosen implementation technique (Section 6.3). This is the continuation of an assumed constant effective velocity within the whole processing interval and thereby it considers a purely linearly moving single point scatterer as illustrated in Figure 7 (Section 3.2). In the case of a confined dominant point scatterer and an idealised propagation channel with a transfer function $|H_{\mathrm{trg}}\left(f\right)|=\mathrm{const}$, this might be a plausible model (Figure 6). The downside is that any deviation from this assumed pure uniform rectangular shape relates to a lower achievable coherent integration gain. Any deviation from a uniform spread will then lead to a suboptimal refocusing that includes parts with less energy and more integrated noise – a kind of ‘collapsing loss’ [13] may occur.

As it has been shown by examples of an actual target response, a practical extended target return might fluctuate even within an occupied bandwidth of just 8MHz in excess of 300ms (Section 5.5). The observable in-band fluctuations raise the question how to properly process and define a kind of wideband or high time-bandwidth product related radar cross section (RCS). The classical ‘reflected power’ to ‘incident power density’ model [13] and the Swerling cases are typically used for confined ‘narrowband’ and mostly monostatic target returns. The target model of extended targets [11] consists contrarily of one or more scattering regions with individually associated complex amplitudes. If a high bandwidth provides a range resolution that is smaller than the target dimension, these scattering regions on a target will become resolved. This has to be addressed in further considerations but the totally distinct time-bandwidth product sets as expressed in Table 1 (Section 2.5) for the same fixed boundary cannot be treated equally. Furthermore, the bistatic geometry might lead to another bistatic RCS definition because the radar return will change for varying aspect angles [11,20]. A joint processing [29,47] applied to extended target returns might however regain some of its lost visibility. It is a subtle but noteworthy difference that very weak extended target responses close to the detection threshold might also look similar to an in-band fluctuating return but this is simply caused by the overlap of present noise. A detailed CEM object and propagation environment modelling might be useful to give more insight to highly fluctuating returns because these are likely caused by a combination of actual target extension, aspect angle changes and multipath propagation.

Last but not least, the effective acceleration of the target return will give an instantaneous Doppler relation (Section 3.3) so that the scale factor s(t) will not anymore be constant within the CPI duration. A time-dependent instantaneous effective velocity yields from Equation (13) with the bistatic range $R_{\mathrm{bi}}$ (Section 2.4)

(31) $$\begin{array}{c} v_{\mathrm{est}}\left(\tau \right)=\frac{1}{2}\;\frac{\partial \;R_{\mathrm{bi}}\left(t\right)}{\partial \;t}{|}_{t=\tau }\approx c\;\frac{1-s(t)}{1+s(t)}\;{|}_{t=\tau }. \end{array}$$

**Figure 15:**
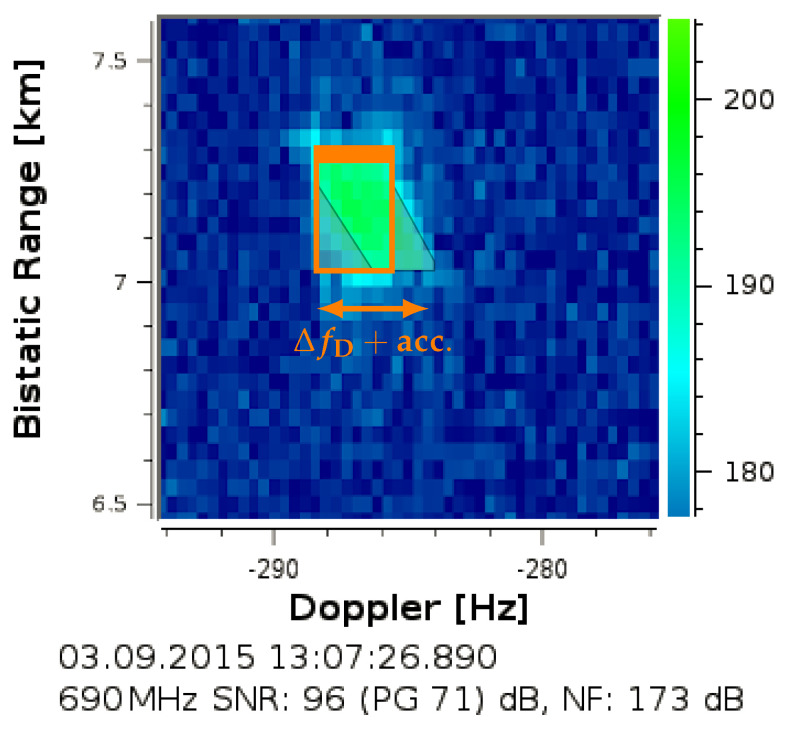
Limit of a fixed time-scale analysis with the example from Figure 6b of slight acceleration, the deviation from a rectangular extent would cause less returned energy and more noise integrated.

Even a slight acceleration would then not only cause less energy—due to parts cut from the rectangular return—but also pure noise being integrated in regions that are no longer inside the otherwise assumed rectangular shaped extension. This is illustrated in Figure 15 for the example of an actual target return. An uncompensated processing will obviously be much worse for stronger acceleration (Section 5.6). This motion influence could however be addressed if the target return acceleration was partly predictable like the change of the effectively measurable velocity in a bistatic radar geometry (Section 2.4). The principle is based on the idea that a constant linear movement with a constant target speed over ground $\left|v_{\mathrm{trg}}\right|$ is still a valid model within the considered CPI duration $T_{\mathrm{cov}}$. Only the effectively measurable velocity $\left|v_{\mathrm{eff}}\right|\leq \left|v_{\mathrm{trg}}\right|$ as expressed in Equation (8) might simply deviate due to the position dependence of the bistatic angle pairs in a bi- or, respectively, multistatic radar geometry. This leads to a geometry-induced acceleration even in the case of a straight-line trajectory without target acceleration. Most compensation methods that were shown in Section 6.3 can however not flexibly account for non-constant and time-dependent scale factors that change within the processing interval.

### 6.5. Remaining Limitations of Velocity Spread Compensation on the Achievable Integration Gain

The evaluation for target detections beyond the time-bandwidth product boundary (Section 2.5) and the maximum unambiguous effective velocity spans of classical batched range-Doppler processing schemes give reasons for optimisations to address the movement implications (Section 4). The batched implementation scheme (Section 2.2) introduced impact is mainly its unambiguously measurable Doppler frequency window that will affect the processing of fast moving targets. This is particularly important for OFDM symbol durations given in the millisecond range in passive radar applications based on digital audio or digital video broadcasting [40] and a block duration $T_{b}=T_{u}$. The fold-over of target returns would hereby give wrong estimates in the Doppler domain as illustrated in Figure 8 (Section 4). A proper identification is then required to associate detections for compensation correctly (Section 6.3) and also to estimate the target speed with its prospective movement for tracking. The remaining Gross Doppler induced frequency sensitivity ${\mathrm{Loss}}_{\mathrm{PC}}$ Equation (18) will need special attention in batched pulse compression schemes because this loss can become severe for a long block duration $T_{b}$ even for small effective velocities [25]. Strong clutter or targets present in adjacent unambiguous windows will defocus but these can still overlay the returns in the currently processed window. Further, an unconsidered range-Doppler straddling loss caused by the temporary displacement between discretised processing bins can impact by several dBs in the quadrature sampled case. The actual amount depends on the return position and the processed waveform (Section 4). This impact also remains if actual small confined target returns are evaluated after the range migration induced spread has been compensated by for example a time-scale analysis approach (Section 6.2). The re-confined returns might still be split between up to four discretised range-Doppler cells if this impact was not considered.

The main drawbacks with regard to any compensation approach are their added computational complexity and the introduced latency. It is in general desired to add just a small latency because any processing delay will enlarge the time delay to obtain a measurement in real-time oriented implementations. If a particular minimal detection range had to be achieved, this would then require even higher coherent integration gains to cope with the added latency. This will in turn likely involve more effective target motion implications. In order to evaluate these influences in greater detail, it is important that the Doppler effect is in fact based on the instantaneous velocity of the target at a particular point in time, i.e., $v_{\mathrm{eff}}\left(\tau \right)=0.5\;\partial_{t}\;R_{\mathrm{bi}}\left(t\right){|}_{t=\tau }$ according to Equation (31) so that it may not be considered as constant over a long processed duration $T_{\mathrm{cov}}$ anymore (Section 6.4). An advanced target motion modelling that is superior to a constant effective velocity would have to include velocity changes (acceleration) and possibly even changes of acceleration (‘jerk’) to avoid de-correlation [20]. It is however unlikely that the target motion can be fully addressed according to a theoretical six degrees of freedom movement in radar detection. Each aspect would open an additional search dimension which can likely not be addressed conclusively in proper processing time. The effective acceleration induced by the radar geometry is however at least partly predictable if the target location and true ground speed have been determined before (Section 6.4). This motion influence could then be addressed by narrowband methods [1,31] for an adaptive processing also after the primary bandwidth-dependent Doppler impact of linear range walk has been compensated. Because in particular an instantaneous acceleration influence on the effective velocity likely increases over time and concurrently with an increased processing duration the resolved velocity cells become smaller, the acceleration poses mainly a limitation for the maximum covered duration $T_{\mathrm{cov}}$. Other target motion considerations include a limited target visibility due to a simple wandering out of the antenna main beam for steered systems or shadowing by terrain and target return fluctuations e.g., due to aspect angle changes in the scattered return and spatial decorrelation [11] or not target related multipath propagation.

### 6.6. Summary

All of the compensation methods shown in Section 6.3 try to address primarily a pure uniform rectangular extent that results from a constant effective velocity with varying computational effort. This is practically limited and it will not compensate actual target return fluctuations and possible deviations for example introduced by motion (Section 6.4). Even if a slight improvement was possible, the summarised influences constrain the maximal realized and achieved coherent integration gain $G_{\mathrm{int},\mathrm{achieved}}$ even below the theoretical maximum of $G_{\mathrm{int},\mathrm{desired}}=T_{\mathrm{int}}\;B_{\mathrm{eff}}$ (7) from Equations (10) and (4) to

(32) $$\begin{array}{c} G_{\mathrm{int},\mathrm{achieved}}\leq G_{\mathrm{int},\mathrm{compensated}}\leq G_{\mathrm{int},\mathrm{desired}}\leq T_{\mathrm{cov}}\;B_{\mathrm{eff}}\leq T_{\mathrm{cov}}\;f_{s}.\; \end{array}$$

The upper bounded consideration in Equation (32) is important for an in-depth performance evaluation by that the products $B_{\mathrm{eff}}\;T_{\mathrm{int}}$ and $T_{\mathrm{cov}}\;f_{s}$ are practically limited. The question how much coherent integration gain can be realized and if a target hit also leads to a better detection and established track depends on many of the described factors. A remaining extension either caused by the resolved target dimension, multipaths or due to straddling losses overlayed by target fluctuations within the bandwidths $B_{\mathrm{eff}}/B_{\mathrm{occ}}$ (Section 5.5) and strong non-stationary clutter will still raise particular challenges. Any concept will need a careful evaluation of the influences present. The possibility to cope with hardware implications like drifts, oscillator stability and phase noise are equally important. These aspects will determine the final detection and localisation capability. However, if the target was ‘visible’ with less coherent gain close to the detection threshold in individual channels, it could be processed non coherently with lower coherent integration gain. This might avoid some of the implications shown.

## 7. Conclusions

The moving target detection becomes severely complicated for a high desired coherent integration gain as it has been highlighted in this publication. The analysis revealed even partly counter-intuitive relations for the processing of the involved high time-bandwidth products and it gave a better definition of the Doppler effect modelling for these cases. A violation of the the stationary boundary can thereby be easily reached even for common processing values often required in coherent radar applications. It was thereby shown that the target decorrelation strength could easily be underestimated if the instantaneously effective bandwidth and batched processing implications are not considered. This can significantly decrease the capability to acquire a target track. The findings were verified with examples obtained from passive radar measurement campaigns and it has clearly shown that the possibility to maintain a high coherent integration gain is worsen against relatively fast moving targets. As it turned out, classical pulse or, respectively, range compression based range-Doppler processing schemes can hardly realize a coherent integration gain of 65 dB against objects with an effective velocity of 200 to 300km/h that is often required in passive radar applications. The practical modelling of a linear target movement is then distinct to a single Doppler frequency displacement and an assumed extent over some range cells. Thereby, even counter-intuitive processing issues related to the bandwidth were identified to be present in a desired coherent multi-channel processing. Further constrains can arise from a batched implementation scheme that might be limited to the long duration of individual OFDM symbols to maintain orthogonality between the tightly spaced sub-carriers for stationary DPI and clutter suppression. Even if time-scale analysis approaches are in principle feasible, these are subject to practical target returns and pose challenges related to their implementability, practicability, the introduced processing effort and latency. Considering the full range of effective aircraft velocities of just 100 to 800km/h, this gives particular challenges to cope with the motion involved implications.

## Figures and Tables

**Table 1:** Different sets before range walk, $v_{\mathrm{eff}}=700\;\mathrm{km}/h,c=c_{\mathrm{em}},$ $f_{s}\approx B_{\mathrm{occ}}$ and ${\mathrm{TB}}_{\max}=58.8\;\mathrm{dB}$.

Time	Bandwidth	Max. Resolution	Frequency	${\mathrm{TB}}_{\max}$
$T_{\mathrm{cov}}$/s	$B_{\mathrm{occ}}$/MHz	$\delta r_{\mathrm{bi}}$/m	$\delta f_{d}$/Hz	dB
1	0.77	194.8	1	58.8
0.5	1.50	100.0	2	58.8
0.1	7.70	19.5	10	58.8
0.05	15.40	9.7	20	58.8
0.02	37.93	4.0	50	58.8
0.01	75.86	2.0	100	58.8
7.59 × $10^{-4}$	1000	0.15	1318	58.8

## Appendix A. Derivation of the Frequency Sensitivity from Equation (18)

The relation shown in (18) can be retrieved by the frequency response similar to an MTI filter. The range dependence vanishes within the guard duration α of a cyclic prefix OFDM signal according to the FT time shift theorem. The following approach considers a single Doppler displacement $f_{D}\left(f_{c}\right)$ by just the carrier frequency $f_{c}$ either after compensation of the occupied bandwidth dependency over $B_{\mathrm{occ}}$ by e.g., the Keystone transform or because the time-bandwidth is sufficiently small (Section 2.5).

Starting with a zero-padded look on the OFDM ambiguity function from [24] with $T_{b}=T_{u}$ yields

(A1) $$\begin{array}{cc} \left|\chi \left(\tau ,f_{D}\right)\right| & =\left|\sum_{i=1}^{\mathrm{noc}}\sum_{k=1}^{\mathrm{noc}}d_{i}d_{k}^{*}\exp\left(j\;2\pi \;f_{k}\;\tau \right)\int_{\tau }^{T_{b}}\exp\left(j\;2\pi \;\left(f_{i}-f_{k}+f_{D}\right)\;\zeta \right)\;d\zeta \right| \end{array}$$

If orthogonality is perfectly fulfilled, values will just result in case of i=k. Therefore,

(A2) $$\begin{array}{cc} \left|E[\chi ]\right| & =\left|\sum_{i=1}^{\mathrm{noc}}\exp\left(j\;2\pi \;f_{i}t\right)\int_{\tau }^{T_{b}}\exp\left(j\;2\pi \;f_{D}\;\zeta \right)\;d\zeta \right| \end{array}$$

which is [24]

(A3) $$\begin{array}{cc} \left|E[\chi ]\right| & =\left(T_{b}-\left|\tau \right|\right)\left|\sum_{i=1}^{\mathrm{noc}}\exp\left(j\;2\pi \;f_{i}t\right)\right|\mathrm{sinc}\left(\pi \;f_{D}\left(T_{b}-\left|\tau \right|\right)\right). \end{array}$$

The signal voltage loss as difference to the optimum has to be squared to get a power factor

(A4) $$\begin{array}{cc} \; & \frac{1}{{\mathrm{Loss}\;}_{\mathrm{PC}}\left(\tau ,f_{D}\right)}={\left(\left(T_{b}-\left|\tau \right|\right)\;\mathrm{sinc}\left(\pi \;f_{D}\left(T_{b}-\left|\tau \right|\right)\right)\right)}^{2}. \end{array}$$

If (A4) is expressed for an overlapped cyclic prefix OFDM symbol train, it is independent of range within the guard duration α. Therefore, using u=max(0,τ−α) instead of |τ| in (A4), the frequency sensitivity loss depends on the Gross Doppler displacement $f_{D}$ as shown in (18)

$$\begin{array}{cc} \; & {\mathrm{Loss}\;}_{\mathrm{PC}}={\left(\frac{1}{\left(1-\frac{u}{T_{b}}\right)\;\mathrm{sinc}\left(\pi \;f_{D}\;\left(T_{b}-u\right)\right)}\right)}^{2}. \end{array}$$
